# A novel speculum-free imaging strategy for visualization of the internal female lower reproductive system

**DOI:** 10.1038/s41598-020-72219-9

**Published:** 2020-10-06

**Authors:** Mercy N. Asiedu, Júlia S. Agudogo, Mary E. Dotson, Erica Skerrett, Marlee S. Krieger, Christopher T. Lam, Doris Agyei, Juliet Amewu, Kwaku Asah-Opoku, Megan Huchko, John W. Schmitt, Ali Samba, Emmanuel Srofenyoh, Nirmala Ramanujam

**Affiliations:** 1 Department of Biomedical Engineering, Duke University, Gross Hall Rm 370, Durham, NC 27713 USA; 2 Center for Global Women’s Health Technologies, Duke University, Durham, NC USA; 3 Duke Global Health Institute, Duke University, Durham, NC USA; 4 Family Planning and Reproductive Health Unit, Greater Accra Regional Hospital, Accra, Ghana; 5 Department of Obstetrics and Gynecology, Duke Medical Center, Durham, NC USA; 6 Department of Obstetrics and Gynecology, Korle Bu Teaching Hospital, Accra, Ghana; 7 The University of Ghana Medical School, Accra, Ghana

**Keywords:** Cancer imaging, Cancer prevention, Cancer screening, Cervical cancer, Diagnosis, Medical imaging, Public health, Biomedical engineering, Applied optics

## Abstract

Fear of the speculum and feelings of vulnerability during the gynecologic exams are two of the biggest barriers to cervical cancer screening for women. To address these barriers, we have developed a novel, low-cost tool called the Callascope to reimagine the gynecological exam, enabling clinician and self-imaging of the cervix without the need for a speculum. The Callascope contains a 2 megapixel camera and contrast agent spray mechanism housed within a form factor designed to eliminate the need for a speculum during contrast agent administration and image capture. Preliminary bench testing for comparison of the Callascope camera to a $20,000 high-end colposcope demonstrated that the Callascope camera meets visual requirements for cervical imaging. Bench testing of the spray mechanism demonstrates that the contrast agent delivery enables satisfactory administration and cervix coverage. Clinical studies performed at Duke University Medical Center, Durham, USA and in Greater Accra Regional Hospital, Accra, Ghana assessed (1) the Callascope’s ability to visualize the cervix compared to the standard-of-care speculum exam, (2) the feasibility and willingness of women to use the Callascope for self-exams, and (3) the feasibility and willingness of clinicians and their patients to use the Callascope for clinician-based examinations. Cervix visualization was comparable between the Callascope and speculum (83% or 44/53 women vs. 100%) when performed by a clinician. Visualization was achieved in 95% (21/22) of women who used the Callascope for self-imaging. Post-exam surveys indicated that participants preferred the Callascope to a speculum-based exam. Our results indicate the Callascope is a viable option for clinician-based and self-exam speculum-free cervical imaging.

*Clinical study registration* ClinicalTrials.gov https://clinicaltrials.gov/ct2/show/record/ NCT00900575, Pan African Clinical Trial Registry (PACTR) https://www.pactr.org/ PACTR201905806116817.

## Introduction

For most women, transgender men, and gender non-conforming persons with a vagina and/or cervix, a gynecologic exam, also known as a pelvic exam, is a regular part of wellness visits starting at the age of 21 until age 65 or later. The internal examination aspect of the pelvic exam traditionally utilizes a metal or plastic duckbill speculum to separate the vaginal canal so a clinician can view the cervix. The speculum-based pelvic exam is performed for various reasons including: (1) cervical cancer screening, i.e. visualizing the cervix and vaginal walls for any malignancy or suspicious pre-cancerous or cancerous lesions and obtaining cell and tissue samples to detect cancerous cells^1^, (2) checking for sexually transmitted diseases, vaginal infections, and abnormal discharge in symptomatic patients^2^, (3) checking cervical dilation during pregnancy^3^, (4) placement and removal of intrauterine device (IUD) as well as monitoring strings for placement verification^4^, and (5) collecting evidence of sexual abuse^5^. While speculum-based exams are one of the most important aspects of reproductive healthcare, it is a large cause of fear of the exam^6–9^.

In the United States, middle-aged African-American women have the highest rates of cervical cancer incidence and mortality at more than double that of their white counterparts^10^. This disparity in cancer incidence and mortality can be partially attributed to a sixfold increase in non-adherence to screening relative to the general population, largely because of the perceived pain of the screening procedure among African American women and the cost of office visits^6^. The supine position a patient is required to be in for the exam, and the form factor of the speculum itself, creates anxiety, fear, discomfort, pain, embarrassment, and vulnerability, which are significant reasons why patients avoid gynecological exams, including the specific speculum-based exams, such as the Pap smear and colposcopy used to screen for cervical cancer^6–9^. One longitudinal study of sexually active young women indicated that women’s perceptions of pain for the speculum during an exam led directly to poor compliance with follow-up procedures after the Pap smear^11^. In Ghana, cervical cancer is the most common cancer in women. The high prevalence has been attributed to inadequate awareness of the disease, limited access to care, fear, and cultural taboos surrounding the exam and reproductive health more broadly^12–15^. These barriers to screening in the United States and Ghana are echoed globally; for example, a study of Australian women’s attitudes towards physician-insertion versus self-insertion of a speculum found that 91% of women (n = 133) preferred self-insertion, and that having another person insert the device caused discomfort, embarrassment, and vulnerability^16^. Further, a study of women in Moshi, Tanzania (n = 354) showed that key barriers to obtaining gynecological exams included concerns about embarrassment and pain due to the speculum, and physician gender^17^. Discomfort from the speculum is particularly exacerbated in patients with vaginismus, a condition involving involuntary tightening of the vagina often caused by sexual abuse^18,19^. Additionally, countries with high sexual violence rates tend to have high rates of cervical cancer incidence and mortality^20–22^.

A second significant challenge in gynecology is providing adequate care to female-to-male (FTM) transgender persons. Over 90% of FTM transgender persons retain their cervices after transitioning and still require comprehensive gynecologic care, including cervical cancer screening^23^. Various studies have shown that FTM persons are less likely to obtain gynecologic care and cite emotional and psychological distress, negative healthcare experiences, mistreatment at health facilities and refusal of services based on their gender identity^24–26^. This is especially problematic given that FTM persons are at a higher risk for undetected HPV infection and subsequent cervical cancer^23^. This is exacerbated in countries where transgender persons face criminalization and are marginalized^27^. Surveys of obstetricians-gynecologists found that only about 30% of respondents felt comfortable caring for FTM patients^28^. Overall, very little information is available to help clinicians improve the gynecologic exam and increase health seeking behaviors for FTM transgender persons.

A third barrier to gynecologic exams stem from travel and time factors associated with visiting a health center^29,30^. Most women have to take time away from work, childcare, housework, and family or business duties in order to go to clinics for screening^31^. Additionally, in certain regions cervical cancer screening clinics can only be found in tertiary institutions, which require significant travel times and higher associated costs, particularly for women in rural areas^32^. Individuals in rural communities often have to travel far distances through unreliable public transportation to reach a health facility. Even if women in rural communities do have access, many health facilities in low-resource areas of the United States and other regions of the world do not have the tools to perform well-established gynecological exams^29,30,33,34^. In high-income areas where institutions exists with the resources to screen for cervical cancer, factors such as work and busy life schedules still deter women from getting screened. A study in Sweden seeking to understand barriers to clinic-based cervical cancer examinations found that more than half of non-compliant study participants stated difficulties in taking time off from work^29^. Creating low-cost yet high-quality medical tools to increase access to lifesaving healthcare assessments such as the gynecological exam could potentially reduce attrition rates for preventive screening and diagnostic tests, particularly among marginalized persons with cervices and/or vaginas^35^.

Another significant, albeit less talked about barrier to seeking gynecological exams, is a lack of knowledge of the reproductive anatomy and reproductive health, which can deter positive health-seeking behavior^36^. In 2014, the American Congress of Obstetricians and Gynecologists (ACOG) and Women’s Health magazine conducted a survey of 7,500 women and found that 64% could not identify a picture of the cervix. The same survey found that 54% of women kept secrets from their gynecologists related to their reproductive anatomy and health^37^. A study of FTM transgender persons found that they had little knowledge of their reproductive health generally, as well as very little information regarding their very unique and specific reproductive health needs and experiences^23^. Educating people about their reproductive health and anatomy has been shown to increase positive health seeking behaviour^36–44^. One strategy to empower women, FTM transgender persons, and non-binary persons with cervices to care and advocate for their reproductive autonomy is to provide self-imaging tools that allows them to visualize, understand, and appreciate their own vaginas and/or cervices.

Although studies have indicated that the speculum causes discomfort, the speculum has not been significantly redesigned in over 150 years despite rapid medical technology advancement. Obviating the need for a speculum by replacing it with a tool that enables increased comfort and the opportunity for self-exams without compromising efficacy, would not only make the traditional gynecological exam less intimidating and painful, but would also provide all individuals, including FTM persons, autonomy over their own pelvic exam, potentially empowering them to seek timely screening. This manuscript describes the potential for a novel technology called the Callascope to address the aforementioned barriers. The aims of this study are to: (1) demonstrate through bench testing methods that the Callascope image quality and application of liquid cervical cancer screening contrast agents is sufficient for cervical imaging and cervical cancer screening; (2) show that the Callascope enables speculum-free, clinician-imaging of the cervix with reduced patient discomfort and increased patient acceptance; (3) assess the feasibility of using the Callascope for speculum-free, self-cervix imaging by healthy volunteers, with low discomfort and high ease-of-use; and (4) validate that results are consistent across multinational clinical settings. Our previous work describes the design exploration and prototyping of the Callascope introducer^45^ . We have also published studies on the development and testing of a low-cost tool, the Pocket colposcope, for cervix imaging^46–48^. However, this work is the first to describe a combined imaging and introducer system for speculum-free cervical imaging with contrast application. Additionally, this is the first clinical study that describes the use of such a device for both clinician-based and self-based cervix imaging in a multi-national setting.

## Materials and methods

### Description of the Callascope

Previous efforts to replace the speculum have replicated its function in a variety of ways including the use of plastic material for the body instead of metal, the use of an inflatable cuff to expand the vaginal walls and introducing a bolus of air to provide a line of sight to the cervix^45^. These solutions function on the premise that the cervix and vaginal walls are viewed from outside the body either with the naked eye or a colposcope, and thus require expansion of the entire vaginal canal, which leads to discomfort. Furthermore, none of these devices have been effective in removing the barriers to screening described above. The Callascope addresses both issues by fundamentally shifting the way the exam is performed with two separate components—a Calla Lily shaped introducer and a slender tampon-shaped camera that fits seamlessly within the introducer.

#### Callascope camera

**Figure 1:**
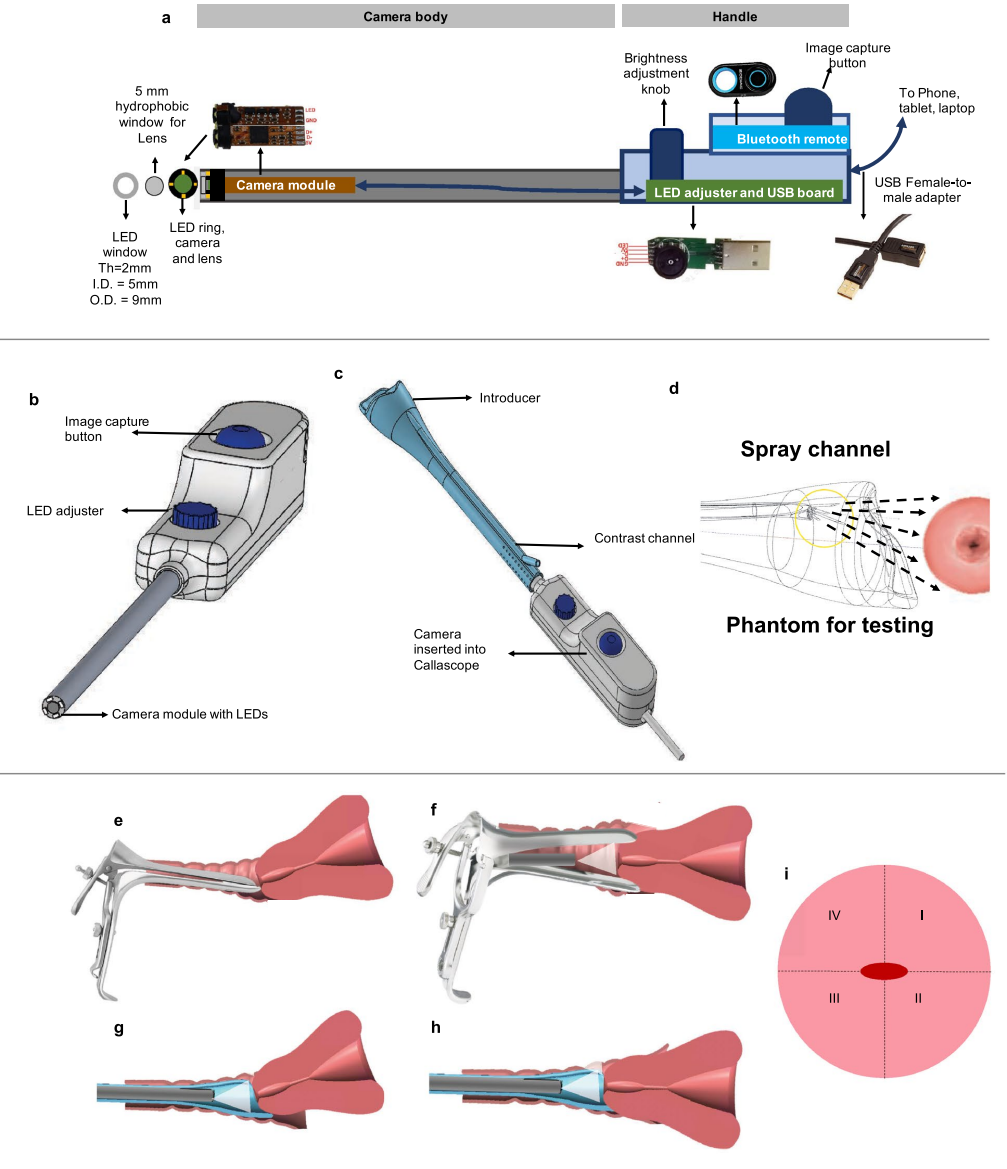
The design of the Callascope promotes visualization of the cervix for diagnostic image capture without the speculum. (**a**) Detailed schematic of Callascope camera. Th: thickness, I.D: inner diameter, O.D: outer diameter. The camera module has a 2MP CMOS sensor, a lens, white illumination LED ring, and a micro processing board. A circular 5 mm hydrophobic window (Edmund optics) is placed over the camera lens and a concentric LED window is placed over the LEDs. A thin layer of black epoxy is applied between the outer edge of the hydrophobic window and the inner edge of the LED window to prevent LED illumination from causing a vignette in the image. The camera module is wired to a micro processing board that controls the LED brightness and connects the camera to a USB board. A female-to-male USB adapter is used to connect the USB board to a computer. A USB adaptor can be attached to enable connection to a phone or a tablet. (**b**) CAD model of the Callascope camera. (**c**) CAD model of the Callascope camera inserted into the introducer. (**d**) Close-up of the spray nozzle of the Callascope showing the fluid distribution path over a cervix. (**e**–**i**) Schematics of cervix visualization used in clinical studies. (**e**) The speculum inserted in closed position into a vagina with a tilted uterus. (**f**) Speculum bills expanded and used to center the cervix for the exam. The Callascope camera, without the introducer was inserted through the speculum to obtain an image with the cervix view enabled by the speculum. (**g**) The Callascope camera and introducer inserted into a vagina with a tilted uterus for cervix imaging without the speculum. (**h**) The Callascope introducer lip centering the cervix to enable imaging with the Callascope camera. (**i**) schematic of a cervix showing the cervix quadrants and os (central red oval) that need to be viewed during cervix examination.

The custom-built camera has a slim tubular body (~ 9 mm diameter, ~ 120 mm length) to fit within the Callascope introducer (described below) and is designed to have a 2–5 Megapixel (MP) CMOS sensor with a lens and a concentric ring of white LEDs at its tip (Fig. 1a,b). The front end of the camera is sealed with a lens and a hydrophobic window, secured with medical-grade epoxy that enables the camera to be water-resistant, and prevents fogging from the humidity of the vaginal cavity. There is a handle at the distal end, which enables brightness adjustment and image capture (Fig. 1a,b). The camera connects via USB to a mobile phone, tablet or laptop. The USB port connection powers the Callascope camera and LEDs; therefore, the device does not require a separate battery or electrical plug-in. Patient data and images are automatically uploaded to a HIPAA compliant cloud-based server, via Amazon Web Services, and are accessible through a web portal. The Callascope allows the user to visualize the vaginal walls as well as the cervix on a portable device. The data collected through the online platform includes information about the person collecting the data, as well as the date and time of collection. The current version of the software also allows an approved remote clinician to log in to review images and make a diagnosis, thus enabling telemedicine. Alternatively, we are exploring methods for these images to be processed using an artificial intelligence algorithm, for risk assessment and to reduce the need for experts^46^.

#### Callascope introducer

The current gynecological exam uses the duckbill speculum to part the vaginal walls to allow for viewing of the cervix from outside the body. In contrast, the slim tubular body of the Callascope and the Calla Lily shaped tip of the introducer can be inserted into the vagina, providing a narrow channel through which a camera is inserted to internally capture a video and/or image of the vaginal walls and cervix (Fig. 1c). The video and/or images are seen in real-time on an external screen such as a cell phone, tablet or laptop computer. In order to provide adequate visualization of the cervix and vaginal canal for diagnosis, the device positions the cervix so that the opening to the uterus (i.e., the os) is centered in the image. The Calla Lily shaped tip of the introducer is the key innovation that allows for speculum-free visualization of the cervix, which typically sits in a tilted position. Once inserted, the asymmetric tip is rotated until the extended lip tilts the cervix into view. In addition to the opening that allows for the camera to be inserted, the introducer has a small working channel along the walls, which can be used for contrast agent application and sample collection during cervical cancer screening procedures (Fig. 1c,d). The distal end (the part farthest from the cervix) of the contrast channel has a Luer lock connector, which couples to a Luer lock syringe for contrast application. The proximal end (the part closer to the cervix) has a nozzle, which sprays the contrast agent uniformly over the surface of the cervix. The nozzle has several holes designed with varying angles to cover a cervix with diameter of 25–30 mm when the camera positioned at a distance of 25–30 mm away from the cervix. Finally, the internal lining of the Calla Lily shaped tip has a reflective coating to ensure uniform illumination and coloration across the entire field of view for imaging. Figure 1e,f demonstrates the insertion of the standard duckbill speculum into the vaginal canal and expansion to bring the cervix into view by an external imaging source. In the clinical studies described in subsequent sections, the Callascope camera (without the introducer) was used to capture the field of view of the cervix, enabled by the speculum. Note that the introducer and camera are separable and the camera can be utilized without the introducer. Figure 1g,h demonstrates insertion of the Callascope (both the camera and introducer) into the vaginal canal and manipulation of the tilted cervix with the curved tip to center the cervix in the field of view. Figure 1i shows a schematic of optimal cervix visualization with all four quadrants and the cervical os in view.

### Callascope image quality and contrast application testing

The resolution, Weber’s contrast and field of view testing as described by Lam et al., 2018 was performed on the Callascope camera and compared to a standard $20,000 standard-of-care colposcope (Leisegang Optik 2)^47^. To test the resolution, both devices were used to image a USAF 1951 resolution target. Resulting images were analyzed using a line scan method with open source Fiji software^49^. The group and element of the smallest resolvable parrallel lines on the resolution target were determined and used to obtain the smallest resolvable feature size from the USAF 1951 resolution target interpretation table. To determine Weber’s contrast, the pixel intensity values of the mock cervix lesion region in white (“*I_max*”) and non-lesion background cervix region in redish pink (*“I_min”*) were determined using the open source Fiji software and used to calculate the contrast with the equation $$\frac{I\_max-I\_min}{I\_min}$$. The field of view was estimated by placing the camera a fixed distance from target objects of different size and determining the largest object that could be fully visualized by the camera. The colposcope and Callascope distances were set to be similar to the working distance used during a cervical exam. The Callascope field of view was 35 mm, whereas the field of view for the standard-of-care colposcope was at 300 mm at 7.5× magnification which is typically used to obtain a full, magnified field of view of the cervix.

To test the contrast application, a printed circular grid 30 mm in diameter (average cervix diameter), was placed over the cervix area 3D printed uterus model, with plastic tubing representing the vaginal canal. The liquid contrast was drawn with a Luer-lock syringe and attached to the Luer-lock connector on the contrast application channel of the introducer. The camera was inserted into the introducer and the introducer lip was placed up against the circular grid. The syringe was then compressed, enabling the liquid to flow through the channel on the introducer and spray out over the grid. Lugol’s iodine, was used to enable staining for visual assessment of coverage. The percentage of surface area stained was calculated. Testing of the contrast application over the grid of a cervix phantom was carried out for 4 application scenarios: 3 ml of contrast agent with a 3 ml syringe, and 3 ml, 2 ml and 1 ml of contrast agent (Lugol’s iodine which is for visualization of cervical disease) with a 10 ml syringe. 3 ml was selected as the maximum amount of liquid to prevent excess pooling, which would obstruct the view of the cervix for imaging. Each was repeated n = 5 times and averaged.

### Clinical investigations in the U.S. and Ghana

#### Overview

This study consisted of two different arms to test feasibility of the Callascope for clinician- and self-imaging of the cervix. Arm 1 recruited patients who were receiving a routine Pap smear or visual exam of the cervix. Clinician-imaging of the cervix was performed using the speculum, and the Callascope introducer for vaginal expansion and cervix manipulation. Arm 2 recruited healthy volunteers in a clinical setting. Volunteers were provided with a Callascope and instructions to perform self-imaging of the cervix. We utilized a mixed methods approach where cervix images were collected with the Callascope, quantitative scores were collected with surveys, and voluntary qualitative feedback was collected via the comments section of the surveys or via audio.

#### Study settings

Both study arms were performed in tertiary health institutions in two different locations: (1) The Reproductive Health Unit at the Greater Accra Regional Hospital (GARH) in Accra, Ghana and (2) the Gynecology Clinic at Duke University Medical Center (DUMC) in Durham, USA.

#### Participants’ eligibility

18 years or older, female, healthy volunteers accepted.

#### Inclusion criteria

Undergoing Pap smear, visual exam of the cervix or colposcopy.

#### Exclusion criteria

Women under the age of 18 (minors), recent episode of bleeding or preterm labor, pregnant women in their 2nd or 3rd trimesters, subjects who were not competent to give consent.

#### Sample size

Ghana (GARH: arm 1 n = 25, arm 2 n = 10 ) and the U.S. (DUMC: arm 1 n = 28, arm 2 n = 12 ). Clinicians (GARH: n = 2, DUMC: n = 1).

#### Disinfection safety procedures

Disinfection of the Callascope prior to use was performed by submersion in 2% hydrogen peroxide for 8 min at room temperature, as recommended for semi-critical devices (which contact mucous membranes or non-intact skin during use)^50,51^. The Callascope camera (which is water-resistant) was also disinfected prior to each use with the same method. The Callascope introducers, which directly contact vaginal mucosa, were discarded after each patient use. A summary of data collected in the two study arms is shown in Table 1.

**Table 1:** Summary of mixed data collected from the international clinical investigations in the U.S. and Ghana.

Study	Method	Data collected
Study 1: Clinician-imaging of the cervix	Pair of cervix images, one using only a Callascope, the other using the Callascope camera through a speculum	Cervix images
	Patient pre-exam survey	Demographics; top features considered for screening (e.g. comfort, cost); initial perception of the speculum, Callascope, and self-screening
	Patient post-exam survey	Pain rating; comparison of speculum and Callascope, qualitative feedback/comments
Study 2: Self-imaging of the cervix	Self-imaging using a Callascope	Cervix images
	Clinician confirmation of cervix visualization during self-imaging	Successful / unsuccessful cervix visualization
	Volunteer pre-insertion survey	Demographics; top features considered for screening; perceptions of the speculum, Callascope, and self-screening
	Volunteer post-insertion survey	Ease of use and comfort level, qualitative feedback/comments

### Ethics approval

The study was approved by the DUMC Institutional Review Board (Pro00008173, NCT00900575), by the Ghana Health Service Ethical Review Committee, and by the Ghana Food and Drugs Association Clinical Trials Department (PACTR201905806116817). All methods were performed in accordance with the relevant guidelines and regulations. All participants provided written informed consent prior to participation.

### Comparison of the field of view and comfort between a clinician implemented speculum-free and speculum-based cervical visualization in patients in two international settings

This investigation was performed in health institutions in Ghana (GARH: n = 25) and the U.S. (DUMC: n = 28). Clinicians (GARH: n = 2, DUMC: n = 1) underwent a 30-min training on the use of the Callascope, which entailed an explanation of the Callascope functionality, the different parts of the Callascope (introducer and camera) and how they connect. Clinicians in the GARH study consisted of 2 reproductive health nurses with over 20 years of cervical cancer screening experience, whereas the clinician in the DUMC study consisted of a Gynecologist with over 30 years of cervical cancer screening experience. The training included a demonstration of a pelvic exam on a cervical exam and Pap smear test trainer (Nasco Life/Form). Clinicians were then asked to assemble the Callascope for use, and complete a pelvic exam with the Callascope on the test trainer to ensure that they could operate the device and visualize the cervix. Post-training, adult female patients undergoing routine Pap smears (DUMC and GARH) or visual inspection of the cervix with the naked eye (GARH only) were recruited by the clinicians. Patients who agreed to participate in the study completed a pre-exam survey to record demographic information as well as document their perceptions of the speculum, Callascope, and clinician vs. self-screening. All study participants received both the control (standard-of-care speculum exam with the Callascope camera) and the investigational device (the Callascope intoducer with the camera inserted in it). Study participants received the control first, followed by the Callascope. This order was used to prevent any reduction in quality of samples obtained as part of standard clinical procedures in which cell samples are obtained. The study was designed with a two-minute delay between insertions. Since standard-of-care colposcopy imaging was not performed during the Pap smear (U.S.) or during visual inspection with acetic acid (VIA) using the naked eye, the Callascope camera, served as the colposcope, and was used in both arms to image the field-of-view afforded by the speculum and the Callascope introducer (the camera was inserted through the introducer in the test arm and through the speculum in the control arm). Participants completed a post-exam survey to assess comfort using a modified version of the 0–10 Universal Pain Assessment Tool, which shows a series of faces ranging from a happy face at 0, or “no pain”, to a crying face at 10, which represents " worst pain possible"^52^. Using the faces and/or the written descriptions the participants selected the face which represented their level of pain during use of the Callascope and speculum. The participants’ overall perception of the Callascope and the speculum exam was acquired using a Likert scale. Image scores of the cervix were calculated by assigning 1 point for visualization of the cervical os and 1 point for each of the 4 quadrants of the cervix visualized. The percentage of patients who had at least 2, 3, and 4 quadrants imaged, were compared for the Callascope introducer and speculum. The percentage of patients for whom the os was visualized was also compared between the Callascope introducer and the speculum.

We tested feasibility of clinician-based imaging with contrast application using the Callascope camera and introducer with a subset of healthy volunteers (n = 5) as part of the DUMC study. Study participants had Lugol’s Iodine applied through the Callascope introducer contrast channel to observe contrast coverage. Despite observed adequate cervix coverage by the contrast, the study was discontinued due to excess pooling, even with 1 ml of liquid. Future studies are exploring methods to increase efficient spray coverage.

### Testing the efficacy of self-examination in female volunteers

For the self-exam, volunteers (with no prior cervical cancer diagnosis) were trained to use the Callascope without a speculum, and to assess ease-of-use and the feasibility of self-imaging without clinician assistance. Volunteers (DUMC: n = 12, GARH: n = 10) were recruited using online and paper fliers, and by word of mouth. Participants completed a pre-insertion survey. At DUMC, participants were given a user kit containing a Callascope, Android phone and charger, Sani wipes, vaginal wipes, lubricating jelly, and a printed user guide. Participants were asked to watch a video tutorial on a custom-built mobile application, which provided information on how to assemble the Callascope and how to use it to capture images of the cervix. Participants then captured an image of their cervix with the Callascope in a private room, which took approximately 5–10 min. Participants inserted the device in a position that they preferred, though we recommended a semi-recumbent or a standing position with one leg raised. We observed that for most women, a semi-recumbent position was preferred. A few women mentioned using the standing position with one leg raised and placed on a ledge, similar to tampon insertion methods. A nurse confirmed whether the images captured were those of the cervix. In the GARH study, due to limited clinic time, participants did not watch the video tutorial, but instead were given the Callascope pre-assembled and connected to a phone. Participants were asked to image their cervix with a nurse present to confirm whether the image was indeed the cervix, a process which took approximately 5 min. After the self-exam, participants were asked to indicate their level of discomfort on a Likert scale with the following options: “No discomfort”, “Slight discomfort”, “Moderate discomfort”, “A lot of discomfort”, and “Extreme discomfort”. They were also asked to indicate how easy or difficult it was to follow the instructions and to use the Callascope to find and manipulate their cervix. The number of women who were able to attain an image of their cervix, as confirmed by the study nurse was recorded.

### Statistical analysis

**Figure 2:**
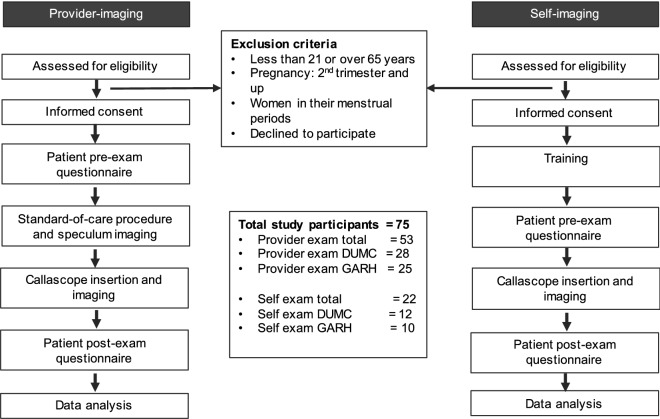
Summary of protocol for clinician-imaging and self-imaging of the cervix. Exclusion criteria and number of participants are shown.

Pre-exam Likert scale responses on willingness, as indicated in Table 2, were analyzed using a non-parametric Wilcoxon signed-rank test (one-tailed, paired, alpha = 0.025). A non-parametric Wilcoxon signed-rank test was used to analyze the post-exam survey data comparing the discomfort level between the speculum and the Callascope (one tailed, paired, alpha = 0.025). A non-parametric Mann–Whitney rank test was used to compare discomfort level between the study sites for both devices (two-tailed, unpaired, alpha = 0.05). Power calculations, using PASS 2015 power program, indicated that a paired, two-sided t-test achieves 80% power to detect an effect size is 0.572 (Cohen’s d) for n = 50 subjects, an effect size of 0.7 for n = 36 subjects and an effect size of 0.8 for n = 28 respectively, at 0.05 level of significance. Since the self-exam was exploratory, no power analysis and statistical tests were performed within each site. Likert scale responses of participant discomfort and ease-of-use were analyzed qualitatively within study sites. Statistical comparison of results obtained at DUMC and GARH was performed using a non-parametric Mann–Whitney rank test (two-tailed, unpaired, alpha = 0.05). Figure 2 summarizes the clinical trial methods described in this section.

**Table 2:** Participant demographics and survey responses.

	Value/options	Clinician-imaging (n = 53)				Self-imaging (n = 22)			
		GARH (n = 25)		DUMC (n = 28)		GARH (n = 10)		DUMC (n = 12)	
Race (pre-survey)	Asian	0		1 (4%)		0		1 (8%)	
	Black	25 (100%)		13 (46%)		10 (100%)		3 (25%)	
	Hispanic (white)	0		1 (4%)		0		0	
	Hispanic (other)	0		3 (11%)		0		0	
	Non-Hispanic White	0		8 (29%)		0		8 (8%)	
	Unknown	0		1 (4%)		0		0	
	Unanswered	0		1 (4%)		0		0	
Age (years) (pre-survey)	Median	40–44		31		30–34		28.5	
	Range	21–59		25–65		21–59		22–56	
	Interquartile range	30–49		28–36					
BMI (pre-survey)	Median	30.1		30.3		27.7		21.7	
	Range	21–49		16–58		22–34		18–38	
	Interquartile range	26–32		25–32					
Number of vaginal births	0	12 (48%)		8 (29%)		6 (60%)		9 (75%)	
	1–2	5 (20%)		12 (43%)		3 (30%)		3 (25%)	
	3–5	8 (32%)		6 (21%)		1 (10%)		0	
	Unanswered	0		2 (7%)		0		0	
Regular use of tampon or menstrual cup	Yes	2 (8%)		10 (36%)		0		9 (75%)	
	No	23 (92%)		15 (54%)		10 (100%)		3 (25%)	
	Unanswered	0		3 (11%)		0		0	
Number of prior speculum exams	≤ 5	25 (100%)		4 (14%)		10 (100%)		6 (50%)	
	> 5	0		19 (68%)		0		6 (50%)	
	Unanswered	0		5 (18%)					
Perception of speculum as a barrier	No-small barrier	23 (92%)		17 (61%)		9 (90%)		10 (83%)	
	Med-large barrier	2 (8%)		9 (32%)		1 (10%)		2 (17%)	
	Unanswered	0		2 (7%)		0		0	

		Spec	Calla	Spec	Calla	Spec	Calla	Spec	Calla
Willingness level to use speculum/Callascope based on appearance	Not–slightly	14 (56%)	1 (4%)	12 (43%)	7 (25%)	7 (70%)	0	9 (75%)	0
	Very–extremely	11 (44%)	24 (96%) 0	16 (57%)	21 (75%)	3 (30%)	10 (100%)	3 (25%)	12 (100%)
	Unanswered	0		0	0	0	0	0	0
	***p-value***	***3e−4***		***1e–2***		***1e−3***		***2e−3***	
Willingness level to have a physician use a speculum/Callascope	Not-slightly	11 (44%)	5 (20%)	12 (43%)	7 (25%)	8 (80%)	2 (20%)	Not asked	
	Very-extremely	14 (56%)	20 (80%)20 (80%)	15 (54%)	21 (75%)	2 (20%)	8 (80%)		
	Unanswered	0	0	1 (4%)	0	0	0		
	***p-value***	***ns***		***2e–2***		***8e−3***			
Willingness level to perform a self-exam with a speculum/Callascope	Not-slightly	18 (72%)	9 (36%)	17 (61%)	12 (43%)	8 (80%)	0	Not asked	
	Very-extremely	7 (28%)	16 (64%)	11 (39%)	16 (57%)	2 (20%)	10 (100%)		
	Unanswered	0	0	0	0	0	0		
	***p-value***	***2e−3***		***3e−3***		***2e−3***			

Likert scale responses are grouped into two.

## Results

### Bench testing demonstrates that the Callascope enables sufficient image quality and contrast application for colposcopy, without the need for a speculum

**Figure 3:**
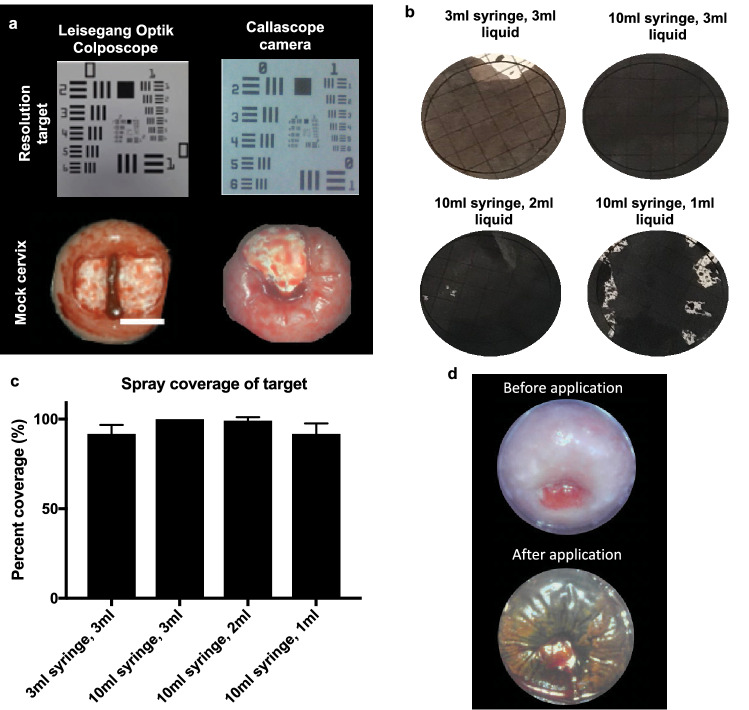
The Callascope is comparable in image quality to a clinical colposcope and provides uniform application of a contrast agent routinely used for visualization of cervical disease. (**a**) Image quality of a $20,000 standard colposcope (Leisegang Optik 2), and the Callascope. The smallest resolvable feature size for both devices are calculated from the resolution target: Standard-of-care Leisegang colposcope = 50.6 µm, Callascope camera = 99.2 μm. Both devices enable visualizing cervix lesion features which tends to be greater than 1 mm (1,000 µm). Weber’s contrast calculated from the mock cervix image are: Standard-of-care Leisegang colposcope = 2.13, Callascope camera = 1.44, however this corresponds to minimal degradation in contrast as is evident qualitatively from the mock cervix images. (**b**) Images of grids show representative distribution of 4 contrast agent application scenarios: 3 ml of contrast agent with a 3 ml syringe, and 3 ml, 2 ml and 1 ml of contrast agent with a 10 ml syringe, with each test conducted with 5 repetitions. 10ml syringe was used to increase the distribution pressure of the liquid contrast agent. (**c**) Bar graphs from contrast application testing (error bars are standard deviations). (**d**) In vivo imaging of a representative cervix stained with Lugol’s iodine (LI) to demonstrate contrast coverage and imaging with the Callascope.

To demonstrate that the Callascope performs equivalently to the standard-of-care clinical colposcope, images of a USAF 1951 resolution target and a mock cervix were obtained using the Callascope (at 4× magnification, at 30 mm working distance), and a standard-of-care colposcope, Leisegang Optik 2 (at 3.75× magnification), as seen in Fig. 3a. The smallest resolvable feature size for the standard-of-care Leisegang colposcope was 50.6 μm, whereas the Callascope camera’s was 99.2 μm. Both devices enable visualizing cervical lesion features, which tends to be greater than 1 mm (1,000 µm). Weber’s contrast was slightly higher for the standard-of-care Leisegang colposcope than the Callascope camera (2.13 vs. 1.44), however, this corresponds to minimal degradation in contrast as is evident from the mock cervix images. The field of view at respective magnification/working distance was 50.6 mm for the standard-of-care Leisegang and 30 mm for the Callascope camera. The cervix diameter typically ranges from approximately 25 mm to 30 mm and thus is within the field of view achieved by either device^53^ A disadvantage of the standard-of-care colposcope is that it is located outside the body, typically at a distance of about 30 cm (300 mm) from the cervix, capturing images including extraneous information like the speculum and vaginal walls.

Testing of the contrast applicator over the grid of a cervix phantom was carried out for 4 application scenarios: 3 ml of contrast agent with a 3 ml syringe, and 3 ml, 2 ml and 1 ml of contrast agent (Lugol’s iodine, which is for visualization of cervical disease) with a 10 ml syringe (Fig. 3b). Administration of 3 ml and 2 ml volume of the contrast agent with the 10 ml syringe provided the most uniform and complete distribution over the grid (Fig. 3c). This is likely due to the higher pressure afforded by the 10 ml syringe. Further, comparable distribution was observed applying the contrast agent Lugol’s iodine, in vivo, through the Callascope channel, for n = 5 healthy volunteers. (Fig. 3d).

### Participants demographics and baseline perceptions of the Callascope vs. the speculum—a two country assessment

After confirming comparable image quality between the Callascope and a standard-of-care colposcope, a two-arm clinical study was performed to assess clinical cervix images capture by a clinician using the Callascope camera with a speculum and with the Callascope introducer, as well as to assess the feasibility of performing a self-exam using the Callascope. This study consisted of two groups of participants: (1) patients undergoing a routine Pap smear or visual exam of the cervix, who then underwent a clinician-based exam where the Callascope camera was used to image the cervix either through the Callascope introducer or through a speculum (to mimic conventional speculum-based visualization); and (2) volunteers in a clinical setting who performed self-imaging of the cervix using the Callascope camera and introducer. Both study arms were performed in tertiary healthcare institutions in two different locations: Greater Accra Regional Hospital (GARH) in Accra, Ghana and Duke University Medical Center (DUMC) in Durham, USA. Participant demographics for clinician-imaging and self-imaging from both DUMC and GARH clinical sites are summarized in Table 2. Additionally, Table 2 includes patient’s baseline perceptions of the Callascope compared to the speculum for gynecological examination. These results were derived from a pre-examination questionnaire. Factors that are known to impede visualization of the cervix, such as high BMI, high number of vaginal births, and age-related vaginal atrophy, were documented in the questionnaire. The median age across sites and studies was within the established age for cervical cancer screening (21–65) and the participants were representative of the population. Median BMI for participants in the clinician-imaging cohort at both DUMC and GARH was ~ 30. For the self-imaging arm of the study, the median BMI at GARH fell within the overweight range, whereas at DUMC it fell within the normal weight range. We enrolled women across a representative range of parities in the clinician-imaging cohort, unintentionally however, most women in the self-imaging cohort had a parity of zero. Most women in the DUMC study had previously undergone more than five speculum-based exams, whereas most women in the GARH study had undergone less than five speculum-based exams. Additionally, we collected information on regular use of tampons/menstrual cups. None of the participants from GARH used tampons or menstrual cups, whereas about 50% of women from DUMC regularly used tampons and/or menstrual cups.

Before participants were shown the Callascope device, most women did not consider the speculum a barrier to being screened for cervical cancer, as most participants indicated they felt there was no other alternative and it was necessary for screening. After being shown the Callascope, participants overwhelmingly preferred the appearance and ergonomics of the Callascope to that of the duckbill speculum. Most women across both study sites reported being very or extremely willing to use the Callascope and not willing or only slightly willing to use the speculum for both clinician-imaging and self-imaging of the cervix.

### Clinician imaging with the Callascope provides comparable field of view and superior comfort compared to clinician imaging with a conventional duckbill speculum

**Figure 4:**
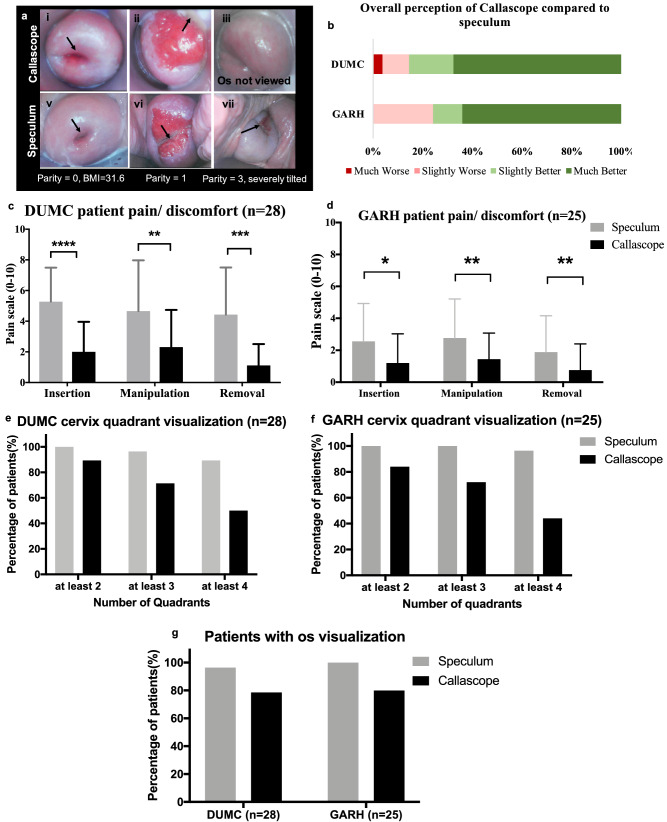
Clinician-imaging of the cervix. (**a**) Representative cervix images obtained using the Callascope camera with introducer by itself and the Callascope camera through a speculum (to mimic a conventional speculum-based colposcopy exam) for cases (i) where the Callascope introducer enabled the full cervix to be seen, (ii) where the Callascope introducer enabled the cervix to be partially seen, (iii) where the cervix was titled away and could not be centered by the Callascope introducer; top and bottom cervix images of each column correspond to the same patient with the top representing images captured through the Callascope introducer and the bottom representing images captured through the speculum. Black arrows indicate the cervical os. (**b**) Patient rating on a Likert scale comparing overall experience with Callascope exam compared to the speculum exam. (**c**) DUMC patient reported pain/discomfort scores for the speculum and Callascope introducer during insertion, manipulation and removal. (**d**) GARH patient reported pain/discomfort scores for the speculum and Callascope introducer during insertion, manipulation and removal. (**e**) Number of cervix quadrants visualized in DUMC patients, for the speculum and the Callascope introducer. (**f**) Number of cervix quadrants visualized in GARH patients, for the speculum and the Callascope introducer. (**g**) Percentage of patients for whom cervical os was visualized for the Callascope and speculum. *P*-values: * ≤ 0.05, ** ≤ 0.005, *** ≤ 0.0005, **** ≤ 0.00005. Error bars indicate standard deviation. Grey bars represent speculum results and black bars represent Callascope introducer results.

Cervix images captured using the Callascope either through the introducer or speculum are shown in Fig. 4a. The overall patient perceptions of the Callascope compared to the speculum after use are shown in Fig. 4b. These figures demonstrate overall higher preference for the Callascope among both patients in DUMC (~ 85%) and GARH (~ 75%). These results were derived from a post-examination questionnaire where participants were asked to reflect on the overall exam, including ease of finding the cervix, discomfort/comfort and image visualization. There was no correlation between regular tampon/menstrual cup use and participants’ response. In addition, patients scored the pain associated with the Callascope and duckbill speculum using a Universal Pain Assessment Tool^52^. Patients provided pain scores for insertion and manipulation of each device to view the cervix, as well as for removal of the device. The Callascope caused significantly less pain than the speculum in both the DUMC and GARH cohorts (Fig. 4c,d).

We also analyzed visualization of the cervix with each device. The cervix is approximately a circular organ, and visualization of cervical lesions are typically marked on pre-determined quadrants of the cervix (Fig. 1i). The number of cervix quadrants visualized by the speculum and Callascope for DUMC and GARH are shown in Fig. 4e,f, respectively. The percentage of patients in whom the cervical os was visualized is also shown in Fig. 4g. Mean visual scores of cervigrams were calculated by assigning 1 point for visualization of the cervical os and 1 point for each of the 4 quadrants of the cervix visualized and averaging these points. The mean scores of cervigrams captured at DUMC were 4.82/5 for the speculum and 3.89/5 for the Callascope; the mean scores of images captured at GARH were 4.95/5 for the speculum and 3.67/5 for the Callascope. The differences in these mean values are not statistically significant. Even though the speculum is the gold-standard, scores of less than 5 for the speculum were due to the cervix being obscured by the vaginal wall between the bills of the speculum. The Callascope does not have bills that push on the vaginal walls, but rather provides a 360-degree expansion of vaginal walls with the asymmetrical curved tip of the introducer. The Callascope enabled visualization of the os in 78.6% of DUMC and 80% of GARH participants. Whereas, the speculum enabled visualization of the os in 96% of DUMC and 100% of GARH. The Callascope enabled visualization of at least 2 cervix quadrants in 89% of DUMC and 84% of GARH participants, at least 3 quadrants in 72% of GARH and 71% of DUMC, and all 4 quadrants in 50% of DUMC and 44% of GARH.

On average clinicians found the Callascope better than the duckbill speculum for manipulating the cervix into an acceptable place for visualization. Two out of three clinicians found the Callascope to be the same or better than the speculum for cervix visualization in their patients (n = 25 patients). One clinician found it to be the same or better than the speculum for 65% of 28 patients and worse than the speculum for 35% of 28 patients.

### Study participants are able to perform self-exams to visualize their own cervices

**Figure 5:**
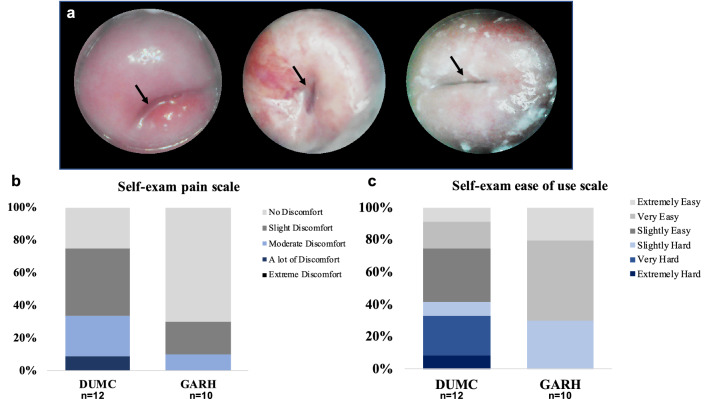
Self-imaging of the cervix. (**a**) Representative images captured by participants of their cervices through self-imaging. (**b**) Particpant-reported comfort level during Callascope use. (**c**) Particpant-reported ease of use of the Callascope to image their own cervix.

Cervix images captured through self-examination are shown in Fig. 5a. A representative video shows how the Callascope is inserted and manipulated by a participant to visualize her cervix as shown in the link included here (https://drive.google.com/open?id=1oA7d1512n4dsQYMDSWVU-RslYtNvytsp). Eleven of 12 participants at DUMC and all 10 participants at GARH were able to visualize their cervix by self-imaging. In a post-insertion survey (Fig. 5b,c), no participant indicated extreme discomfort, and 70% of DUMC and 90% of GARH participants indicated no or slight discomfort. Most participants at DUMC (~ 60%) and GARH (~ 70%) found the Callascope easy to insert and use to find and image their cervices. Only one DUMC participant reported on the survey that it was extremely hard to use. We included women and clinicians from both a high-income and a lower-middle income country (US and Ghana) and found no significant differences in outcomes. The results from the self-imaging study agree with those from an earlier feasibility study conducted with an earlier version of the Calla Lily introducer where over 90% of participants in the earlier feasibility study preferred the Callascope to the speculum, and 83% were able to visualize their cervix^45^. There was no correlation between regular tampon/menstrual cup use and participants response. Post-study, participants provided optional written/audio comments, with representative comments in the pargaraphs below.

“The pictures were, well, sort of fascinating to see yourself the way a Doctor or a medical student sees other people’s bodies. Some Other feeling you know? Some feeling of alienation in looking at myself in terms of such a physical view that is so different than self-image. Yet also it's just nice to be able to really access your own body visually… It probably left me more motivated to undergo gynecological exams if recommended.” – Self-exam study participant.

“I'm a cis female in my late twenties with an IUD, so I've had several gynecological exams over the years and I have been on the receiving end of a speculum and so I guess I was expecting this to be a similar experience, very clinical and impersonal in a way. I think when I think of female reproductive anatomy I think of that it's enigmatic, that it's hidden from us physically. We don't have a lot of external genitalia, but it's also kind of hidden in the way that we don't talk much about vaginas and uteruses and cervices. So, this was kind of an eye-opening experience to be able to visualize something that is so intrinsically a part of me that I have never seen in this way before. It was much more personal and fascinating than I expected it to be.” – Self-exam study participant.

## Discussion

The Callascope has the potential to offer an alternative to the standard duckbill speculum for clinical exams, which like the self-breast exam, will allow individuals with cervices to perform basic cervical exams in their own homes. Since it enables visualization of the cervix, the Callascope can potentially also be used by individuals at home for self-imaging to detect cervical cancer, infections, locating intrauterine device (IUD) strings, and labor dilations. Even though the studies described here were performed in persons identifying as women/female, future clinical studies with the Callascope could specifically be used with FTM transgender persons and non-binary conforming persons with cervices. This device opens new opportunities for self-monitoring in the gynecologic realm and clinician monitoring without the need of the duckbill speculum, which could address loss to follow-up, stigma, and other barriers that prevent positive health seeking behavior. The Callascope device is part of a broader paradigm shift where medical technologies can both improve clinic-based medical care and gives individuals with cervices greater control and information at home.

To date, studies of self-screening for cervical cancer have primarily focused on the acceptability and feasibility of HPV self-sample collection methods, where individuals insert a brush into the vagina and rotate it to acquire samples for HPV testing. Acceptability of these studies has been measured by using scores of discomfort, pain, embarrassment, and privacy. Although physician-collected HPV samples were initially viewed as the gold standard, large-scale studies have shown that women are capable of collecting viable samples for HPV screening, and this approach is currently recommended in several resource-limited settings^54–56^. HPV self-testing to screen for cervical cancer has proven successful and gives women privacy and ownership of a crucial part of the gynecological exam^35^. We expect the same trend for self-imaging of the cervix. Even if women have access to HPV self-testing, follow-up cervical visualization is often needed to improve screening specificity and triage HPV positive women without overburdening local health facilities, particularly in low-and-middle income countries. Self-HPV testing combined with self-visualization of the cervix using the Callascope would give women control over the entire screening process and would allow approximately 98 out of 100 women to complete care at home (only 20% of people screened have high-risk HPV^57–59^ and only 10% of people with high-risk HPV develop persistent infections that make them susceptible to cervical cancer^60–62^). In addition, our group is developing algorithms for automated colposcopy interpretation, which would augment traditional colposcopy readings by healthcare experts. Preliminary results have shown diagnostic accuracy on par with expert colposcopists^46^. Combining automated diagnosis following self-imaging would truly transform the paradigm of gynecologic exams.

We demonstrated the feasibility of imaging the cervix without the speculum both by clinicians and, importantly, by the patients themselves. For the clinician-imaging study, the Callascope introducer enabled visualization of the os in 78.6% of DUMC and 80% of GARH participants. Whereas, the speculum enabled visualization of the os in 96% of DUMC and 100% of GARH. Most women for whom the Callascope failed either had higher BMI, were multiparous, had given birth in the past 3 months, or had a combination of all or some of these factors. These failures occurred due to two factors: (1) the length of the Callascope introducer being insufficient to reach the cervix in women with a higher BMI, and (2) the opening of the Callascope introducer tip being insufficient to fully encompass all quadrants of the enlarged cervix within multiparous or post-natal women. In women who fall under these two categories a larger speculum was used, but this limitation could be overcome in the future by using different Callascope introducer lengths and diameters of the Calla Lily shaped opening. In other words, just as there are multiple sizes and lengths of speculums there could be Callascope devices with different viewing areas and lengths for use on patients of different BMIs, ages and parities. In order to make the cervix area visualized by the Callascope as good or better than the speculum, we will integrate improvements to the design, by exploring alternative tip sizes and lengths to account for women with higher BMIs and multiparity. For the self-imaging study most participants at DUMC (~ 60%) and GARH (~ 70%) found the Callascope easy to insert, find and image their cervices. Only one DUMC participant reported on the survey that the Callascope was extremely hard to use. The greater ease of use indicated by participants at GARH versus DUMC may have been due to the difference in how instructions were provided. At DUMC instructions were provided using a short video tutorial and a printed user guide, after which participants were left on their own whereas at GARH instructions were given verbally by a healthcare clinician while participants used the device.

The primary aim of the paper is to demonstrate the feasibility of imaging the cervix without the speculum either by a provider, or by the patients themselves. Though the clinical studies did not include contrast application of acetic acid or Lugol’s iodine to highlight suspicious lesions for screening and diagnosis, we did show on a small subset of our healthy volunteers the distribution area of contrast (Lugol’s iodine) on the cervix when applied using the Callascope contrast channel. Future studies will include a randomized clinical trial, in which Lugol’s iodine and acetic acid contrast agents are applied specifically to patients getting diagnosed for cervical cancer, and on whom histopathology is performed as part of the standard-of-care diagnosis procedure. This would enable us to perform an image concordance study to determine accuracy of clinician diagnosis with the Callascope versus with the standard-of-care speculum and colposcope, with pathology as ground truth. These images would also provide input for a machine learning algorithm to assess feasibility for automated risk assessment^46^. Other limitations include the small number of clinicians who used the device, the non-randomized nature of the study and the small sample size of patients and healthy volunteers. Future studies will seek to enroll a statistically significant clinician sample size, utilize a randomized clinical trial design and enroll a higher number of patients.

The Callascope is extremely well-suited for use in educational initiatives to improve women’s awareness of their vaginal and cervical anatomy. Demystifying the cervix for women by having them see this vital organ and having them engage with their own bodies has the potential to shift the narrative around reproductive health in a positive way. When users discover their cervices, they will likely be more comfortable talking about their reproductive health. This increased knowledge may improve their awareness of the importance of cervical cancer screening and overall sexual and reproductive health. This is expressed in representative comments participants provided about their experience with the Callascope.

We demonstrate in this manuscript the potential for a novel technology called the Callascope to address the major barriers to gynecological exams. The device has been designed to combine and replace the colposcope and speculum, providing essential information for effective visual examination of the cervix, while reducing the associated discomfort/pain. This technology replaces the speculum with a Calla Lily shaped introducer. Visual inspection with acetic acid with the naked eye (VIA) used in low and middle income settings and/or the traditional high-end colposcope (low power microscope to magnify the cervix), have been replaced with a tampon-shaped light source and camera which allows women or clinicians to capture a magnified image that is then sent to a cell phone or tablet. Furthermore, the introducer encases a working channel, which allows for contrast agent application—an important feature for cervical cancer detection. We demonstrated that the image quality of the Callascope is comparable to a high-end standard colposcope through a variety of image quality testing methods. Through studies in both the United States and Ghana, we demonstrate the effectiveness of the device for clinical use both to visualize the cervix and to perform self-exams with minimal training across a diverse population of women. Moreover, results indicate that women unequivocally prefer the Callascope over the traditional speculum.
